# Keyword Index for Volume 95

**DOI:** 10.1038/sj.bjc.6603541

**Published:** 2006-12-12

11q13 1432

^18^Fluorodeoxyglucose SPECT 1424

18q 1562

1p/19q loss 1424

^201^Thallium SPECT 1424

5-aza-2′-deoxycytidine 1701

5-fluorouracil 13, 35, 131

5-FU 607

accidental poisoning 649

acquired immunodeficiency syndrome (AIDS) 642

activity 13

acute lymphoblastic leukaemia 1537

adenocarcinoma 118

adenovirus 555

adhesion 1497, 1545

adipose tissue 1028

adjuvant therapy 260, 266, 817, 1195

advanced breast cancer 667, 794, 1161

advanced gastric cancer 1642

advanced melanoma 581

aetiologic heterogeneity 123

aetiology 1269

affymetrix SNP array 1415

Africa 355

ageing 520

age-period-cohort analysis 398

age–period–cohort models 1269

Akt 1653

alcohol 378

amplicon 1439, 1689

anaemia 13, 1274, 1467

angiogenesis 1038, 1131, 1371, 1396

angiotensin converting enzyme (ACE) 67

angiotensinogen 67

anthracycline 571

antiangiogenic tumour therapy 272

anticancer drug cytotoxicity 906

antifolates 289, 677

antioxidant defence 525

antitumoral drug 197

antivascular agents 722

anus neoplasms 87

anxiety 435

apoptosis 35, 49, 282, 520, 869, 961, 1114, 1348, 1696

aromatase inhibitors 661

array-CGH 1439

Ashkenazi 1448

aspirin 1277

AT1, AT2 receptors 67

autocrine 322

axillary dissection 811

*β*-catenin 889

basal phenotype 616

Bcl-2 869, 1244, 1653

BCR/ABL 775

BER 239

biliary tract neoplasms 848

bioactive lipid signaling 1131

bioinformatics 425

biomarker variability 42

bioreductive drugs 1229

birth weight 1603

bisphosphonate 1354

bladder 1415

bladder cancer 289, 1234, 1354, 1455

body mass index 366

body weight 153

bone 782

bone markers 506

bone metastases 506, 1354

borderline tumors 1092

Borrmann 744

BRCA 914

BRCA1 515, 757, 1108

BRCA1 and BRCA2 801, 1448

BRCA2 515, 757

breast cancer 67, 107, 123, 339, 347, 385, 390, 435, 525, 616, 642, 653, 674, 683, 757, 801, 811, 914, 974, 1056, 1291, 1334, 1362, 1404, 1410, 1448, 1626, 1683

breast carcinoma 62, 393, 506

breast conserving surgery 1265

breast MRI 801

breast neoplasms 1689

breast screening 62, 1265

Burkina Faso 355

cadherin 1258, 1367

CagA 639

calcium 1582

Calman-Hine 979

camptothecins 906

cancer cachexia 1028

cancer incidence 233

cancer proteomics 425

cancer registration 593, 1576

cancer risk 986

cancer screening 112

cancer survival 146, 1576

cancer-specific survival 1076

cannabinoid 197

cap-dependent translation 955

capecitabine 27, 1195

carboplatin 1309

carcinoembryonic antigen (CEA) 218, 674

carcinoembryonic antigen-related cell adhesion molecule 6 (*CEACAM6*) 532

carcinoma of unknown primary 1309

carcinomas 1670

cardiotoxicity 571

carotenoids 406

case–control study 1288, 1586

caspase activated DNase 1696

caspases 49, 282

castration resistance 767

CD105 1683

CD24 339

CD70 298

CDKN2A/p16 1432

CEA cell adhesion molecule (CEACAM) 218

C/EBP*α* 1028

cell blocks technique 204

cell cycle 555, 1348

cell cycle arrest 869

cell cycle regulation 314

cellular differentiation 307, 485

cellular senescence 496

central nervous system 416

cerulenin 869

cervical adenocarcinoma 331

cervical cancer 226, 385, 1250, 1459

cervical intraepithelial neoplasia (CIN) 1459

cervical neoplasia 96, 1593

cervical screening 56

chemokine 210

chemoradiation 260

chemoradiotherapy 561, 710

chemosensitivity 1424

chemotherapeutic drugs 1038

chemotherapy 27, 266, 463, 485, 848, 862, 966, 1114, 1142, 1334, 1626, 1632

childhood cancer 571

children 102, 416, 571, 991

China 96, 1593

cholangiocarcinoma 532

chromosome arm 3q 331

chronic myeloid leukaemia 1136

cigarette smoking 374

cisplatin 475, 822, 1005, 1348, 1648

client preferences 1448

clinical practice guidelines 1490

clinical resistance 955

clinical services 974

clinical trials 1155, 1459, 1483

clodronate 272

*Clostridium* 1212

CML 775

cohort studies 112, 363, 366, 374, 385, 639, 934, 1277, 1280, 1579

colon 1415

colorectal adenocarcinoma 1637

colorectal cancer 6, 27, 35, 131, 239, 735, 841, 889, 921, 928, 979, 1047, 1101, 1239, 1277, 1321, 1367, 1419, 1525, 1562

colorectal liver metastases 21

colorectal neoplasms 13, 1195

computerised decision support 1490

conjugation 1212

construction 102

continual reassessment method 253

copy number 1087, 1415

core biopsy 62

cost analysis 6

cost-effectiveness 27, 801

COX-2 139, 1234

CPT-11 710

C-reactive protein 1076, 1234

cremophor-free 729

CRP 782

cryo ablation 896

c-Src 1410

CTLA-4 896

cured cells 1020

CXCR4 210

cytokeratin 20 (CK20) 218, 1258

cytokines 1568

cytology 56, 1070, 1250

cytotoxic T cell 1202

cytotoxicity 1038

D2-40 1362

Danish 146

DaunoXome 571

DCI cases 1576

DCIS 1410

delay 835

deletion 548

dendritic cell 896

deprivation 944

DES 107

desmocollin 1367

desmosome 1367

diagnosis 1321

diet 406, 1582

dietary factor 1586

digestive cancer 944

dihydropyrimidine dehydrogenase 607

discrete choice experiment 1448

distance 944

distant metastases 710

DNA damage 520, 1514

DNA fragmentation factor 1696

DNA methylation 541

DNA ploidy pattern 817

DNA repair gene 561

DNA topoisomerase 1 906

docetaxel 457

doxorubicin 282, 1342, 1663

drug combination 289

drug delivery system 601

drug resistance 961, 1537

*E1A* 555

E-4IB isothiocyanate 1348

early detection 331

early onset 1288

early-onset colorectal cancer 752

economic evaluation 1195

EGF 164

EGFR 172, 1070, 1390, 1483, 1525

EGFR targeting 722

elderly 1309

EMAP-II 735

EMMPRIN 1371

endocrine therapy 661

endoglin 1683

endometrial cancer 266, 385, 642, 1555, 1586

EPIC 406

epidemiology 87, 96, 102, 112, 118, 123, 649, 986, 1280, 1579, 1582, 1593

epidermal growth factor receptor (EGFR) 998

epigenetics 541

epirubicin 1005

epistasis 525

epoetin beta 1467

equivalence 435

*ERBB2* 1689

etiology 1274

Ets transcription factor 1404

Europe 378

ewing tumour 1326

excess cases 401

exclusive chemo-radiotherapy 705

exemestane 153

expression profiling 1092

extensive-stage small-cell lung cancer 1648

extrapolation 398

extraversion 146

familial 974, 1288

familial cancer 435

familial risk 1291

Fas 1244

fatty acid synthase 869

feasibility study 853

fibroblast growth factor receptor 4 1455

fibrosarcoma cells 1114

fine-needle aspiration cytology 62

fingerprint 1537

first-line therapy 131, 788, 998

follow-up 21

formulation 729

forskolin 775

G1-phase cyclin-dependent kinase 1514

galectin-3 204

gastrectomy 940

gastric cancer 118, 159, 406, 445, 639, 1504

gastric carcinoma 1371

gastric/GEJ cancer 450

gefitinib 172, 722, 998, 1070, 1390, 1483, 1504

gemcitabine 260, 289, 532, 587, 848, 1309

gene amplification 1432

gene expression 80, 928, 1056

gene polymorphisms 906

gene–gene interactions 525

genetic and epigenetic alterations 1101

genetic counselling 435, 1448

genetic susceptibility 1689

genetics 541, 1288

genital warts 1459

genotypes 355

germline mutations 752

gestational trophoblastic disease 1145

glioblastoma 862, 1155

glioblastoma multiforme 197

glioma 862

glioma cells 869

glucocorticoid 1537

green tea 371

growth 1603

GSK-3 1653

HapMap 1689

head and neck cancer 1432

health services researches 593

height 366, 1603

*Helicobacter pylori* 639, 744

hepatectomy 1050

hepatitis C virus 1598

hepatocellular carcinoma (HCC) 210, 853, 1050, 1379

HER2 1404, 1410, 1504

HER 2-positive metastatic breast cancer 788

hereditary cancer 1678

HIF-1 1

high risk 801

high-grade gliomas 991

histamine 1663

histology 123, 1180

HIV 1598

HIV-1 355

HIV-1 protease inhibitor 1653

HNPCC 1678

hnRNP K 921

Hodgkin's lymphoma 378

homoharringtonine 253

horizontal gene transfer 1696

hormonal therapy 153

hormone-refractory prostate cancer 457

HPV 355, 1384, 1432

HPV detection 56

HPV prevalence 226

HPV vaccine 226

human immunodeficiency virus (HIV) 642

human papillomavirus 96, 331, 1250, 1459, 1593

hybrid capture 2 1384

hyperbaric oxygen 862

hypermethylation 1108, 1701

hypotonic cisplatin treatment 717

hypoxia 307, 735, 928, 1013, 1212

ICD-10 649

ICD-9 649

ifosfamide 1342

imaging 21

imatinib 1013

immortalisation 496

immunohistochemistry 204, 322, 347, 616, 634, 921, 1410

immunotherapy 247, 1167, 1202, 1244, 1474

incidence 87, 91, 416, 1269

incidence trends 398

indoleamine 2,3-dioxygenase (IDO) 1555

inducible gene expression 289

induction chemotherapy 470, 705

infant 1274

infection 102

inflammation 1568

ING2 80

innate immunity 247

inoperable pancreatic cancer 1474

insulin receptor substrate 1220

Insulin receptor-isoform A 172

insulin-like growth factors 112, 1220

integration 1384

interferon-*α* 463

interleukin-10 1076

interleukin-2 463, 1167

interleukin-6 1076

internalisation 298

interphase fluorescence *in situ* hybridisation 331

interstitial fluid pressure 1013

intracellular activation 189

intrapleural chemotherapy 717

invasion 1396

irinotecan 131, 587, 1648

irinotecan-resistant 1637

Ki-67 1234

kinase inhibitors 955

Korea 639

LABC 1005

laparoscopic 6

laryngeal squamous cell carcinoma 314

Laurén 744

leptomeningeal dissemination 991

leucovorin 159

leukaemia 102, 253, 775, 1274

level-I dissection 811

level-III dissection 811

lipid 153

lipodomics 1131

liposomes 272, 822

liver 210, 1545

liver metastasis 1504, 1562

localised prostate cancer 1186

LOH 1562

loss of heterozygosity 515, 541

loss of imprinting 541

low-grade gliomas 1062

low-grade squamous intraepithelial lesions 1384

luminol 189

lung cancer 139, 607, 677, 1013, 1070, 1280, 1288

lung neoplasms 75, 1483

lymph node metastasis 75, 634, 1362, 1611

lymph nodes 841

lymphadenectomy 699

lymphangiogenesis 1362, 1611

lymphatic and vascular invasion 445

lymphatic endothelial cell proliferation (LECP) 1611

lymphatic vessel density (LVD) 1611

lymphocytes 520, 1545

lymphocytes apoptosis 735

M30 Apoptosense 42

M65 Elisa 42

macrophage depletion 272

MAD2 475

magnetic resonance imaging 416

malignant melanoma 91

malignant morphology 889

malignant pleural effusion 717

mammalian target of rapamycin (mTOR) 955

mathematical biology 1136

matrix metalloproteinase 7 (MMP7) 218

matrix metalloproteinases 506

MEK 475

melanoma 80, 181, 496, 829

mesothelioma 649

meta-analysis 112, 139

metallothionein 339

metastasectomy 691

metastasis 210, 425, 1396, 1419

metastatic renal cancer 463, 691

metastogenic phenotype 1101

methotrexate 1537

*MGMT* 1155

microarray 1092

microRNA 1415

microsatellite instability 1239

microvascular endothelium 1497

midostaurin 829

migration 1545

minichromosome maintenance proteins 314

minodronic acid 1354

mitochondria 1529

mitochondrial DNA 1087

mitomycin C 1229

mitosis–karyorrhexis index (MKI) 49

mitoxantrone 457

molecular subtypes 1334

monoclonal antibody proteomics 921

mortality 91, 107, 416, 940, 1321, 1632

motility 164, 1220

*MSH6* 752, 1678

MSI 1678

mTOR 1148

mucin 2 (MUC2) 218

multidisciplinary teams 979

multikinase inhibitor 581

multilevel Cox model 944

multiple basal cell carcinoma 548

multiple myeloma 782, 961

multiple primary malignancies 986

multiple tumours 390

muscle-invasive bladder cancer 561

mutations 757, 914, 998, 1070, 1390, 1529

MUTYH 239

*MYH* 239, 1239

N2-disease 470

*N*-acetylcysteine 35

naevus 496

nasopharyngeal neoplasm 1269

NBCCS 548

NCI-60 panel 906

NDRG1 307

neoadjuvant 710

neoadjuvant chemoradiation 1174

neoadjuvant chemotherapy 1683

neoplasms 416, 1180, 1579

nephrectomy 1076

neuroblastoma 49, 879

neuroendocrine carcinoma 1148

neuroleptic medication 934

neuroticism 146

NF1 233

NF-*κ*B 247

NGF 322

nitroreductase 1212

NK105 601

non-Hodgkin's lymphoma 1598

non-small cell lung cancer (NSCLC) 470, 717, 817, 822, 966, 998, 1142, 1653

nonsteroidal anti-inflammatory drugs 363, 1277

normal breast 515

Norway 366

novel therapy 961

NQO1 1229

NQO2 1229

nutritional assessment 431

octreotide 853

oesophageal cancer 322, 634, 705, 835, 1174

oesophagectomy 940

oesophagus 118, 1180

oestrogen receptor alpha 339

oestrogen receptor-*β* isoforms 616

oestrogens 107

oligodendroglioma 1424

oncocytic cells 1529

oncogene 1439

oral cancer 363, 1396

oral contraceptives 385

orotate phosphoribosyl-transferase 607

orthotopic model 1354

OSI-7904L 450

osteopontin 634

osteosarcoma 1603

outcome 1326

ovarian cancer 385, 627, 642, 757, 1092, 1314

ovarian carcinoma 699, 1087

overdiagnosis 401

oxaliplatin 131, 848, 1195

oxaliplatin-resistant 1637

P14^ARF^ 1670

p16 496

P16^INK4A^ 1670

p27 regulation 1514

p53 35, 49, 496, 627

p53 mutation 1701

p53 target gene 1701

p75NTR 322

paclitaxel 159, 601, 729, 1005, 1642

palliative care 593, 966

pancreatic cancer 260, 307, 587

PATCHED 548

pathologic tumour-node-metastasis (pTNM) stage 1050

paxoral 794

PBSC 1326

PEA3 1404

pemetrexed 677

perceived needs 667

periostin 1396

persistent infection 1020

pharmacodynamics 450

pharmacokinetics 450, 677, 729

phase I 253, 571

phase II 450, 794, 1148

*Phellinus linteus* 282

phosphorylated EGFr 1525

photodynamic therapy 189

phototherapy 485

PI3K/Akt, drug resistance 1504

pilot clinical study 197

place of death 593

platinum complex 1514

pleural effusion 1390

PNA-LNA PCR clamp 1483

podoplanin 1362, 1611

polycomb 1202

polymer micelle 601

polymorphism 1047

population mixing 102

population-based 363, 393, 653, 934, 986, 1296

porphyrin 485

positron emission tomography 1174

PP2A 775

PPAR*δ* 889

PPAR*γ* 879

prediction 691

predictive markers 1683

preoperative diagnosis 204

primary health care 974, 1321

PRL-3 347

probability model 757

profiling 1439

prognosis 339, 506, 627, 634, 691, 835, 1062, 1081, 1371, 1419, 1455

prognostic factors 139, 347, 445, 782, 1186, 1296, 1555

progression 1371

progression-free survival (PFS) 1555

proliferation 616, 1081, 1220

prolonged temozolomide 1155

prophylactic vaccine 1459

(pro)renin 67

prospective cohort study 146, 371

prospective studies 233, 1277

prostate 1415

prostate cancer 164, 282, 371, 398, 401, 425, 767, 1081, 1582

proteasomes 961

protein degradation 961

protein kinase C 829

protein level 744

proteome 1379

proteomics 298

pS2 339

pS6 1148

PSA 401

pseudomyxoma peritonei 1258

PTP4A3 347

puberty 1603

pyrrolidinedithiocarbamate 35

quality of life 667, 683, 966, 1626

quantitative reverse-transcription polymerase chain reaction (QRT-PCR) 1062

quantitative RT–PCR 42

Rac 1081

radiation effects 390

radiation therapy 862

radiofrequency ablation 896

radiosensitiser 601

radiosensitivity 1

radiotherapy 266, 390, 1142

radon 1280

randomised clinical trial 266, 699

randomised controlled trial 433, 811, 817, 1005

randomised discontinuation trial 581

rapamycin 955

real-time PCR 1568

receptors 853

rectal cancer 710

regional treatment 1663

registries 653

regulatory T cell 896, 1202

relative survival 1296

renal cancer 1076, 1167

renal cell carcinoma 298, 374, 1244

reovirus oncolysis 1020

resistance 172, 661

resistance mechanisms 955

response 1174

retinoids 463

retinol 406

risk 363, 393, 914, 934, 1047

risk factors 642

road distance 944

ROS 1497

rosiglitazone 879

routine statistics 1576

RT–PCR 1326

rural 102

S-1 1637, 1642

safety 729

salvage surgery 1342

scintigraphy 853

Scotland 87

screening 801, 1250

screening mammography 1265

second cancer 393

second primary cancer 1142, 1291

secondary dissemination 991

SELDI 1379

selenium 674

sensitivity 1334

serum 1379

SET 775

sexual function 1626

signal transduction inhibitors 661

signalling adapter protein 1419

SIL 355

single nucleotide polymorphism 561, 1455, 1689

sirtuins 1056

Six1 1050

Smad4 1562

small intestinal cancers 1296

social class 1180

socio-economic deprivation 940

socioeconomic position 653

soft-tissue sarcomas 986, 1342, 1663

somatostatin 853

Sorafenib 581

specialisation 979

S-phase fraction 314

sphingosine-1-phosphate 1131

SPI-77 822

squamous cell carcinoma 87

SREBP-1c 1028

stability 42

stage migration 841

staging 653, 1180

standard gamble 683

STAT3 164

*STK15* 1047

stopping rules 253

stress 1579

stressful life events 1579

supplements 1582

supportive care 667

surgery 6, 699, 782, 1167

surgical resection 835

surgical trauma 1497

survival 91, 139, 561, 744, 966, 1050, 1145, 1167, 1234, 1314, 1467

Switzerland 1598

systematic review 139, 457

systems biology 425

T cells 181

t-AML 1108

tamoxifen 118, 153

targeted cancer therapy 1136

targeted drug delivery 189

targeted therapies 131, 661, 691, 767, 1525

TAV 1663

tegafur 27

telomerase 331

telomerase activity 1250

telomerase vaccine 1474

temsirolimus 1148

TGCT 475

The Netherlands 374

three-week schedule 1648

thymidylate synthase 607, 928

thyroid 1670

thyroid cancer 204, 366, 1529

Tiam1 1081

timing 835

TIMP-1 1114

tissue microarray 80

T-lymphocytes 1234

tobacco 378

tocopherol 406

toll-like receptors 247

topoisomerase II 1038

topoisomerase II*α* 1334

toxicity 853

*TP53* 1432

*TP53* mutation 1455

Tp73 isoforms 1062

transcription 555

transferrin 189

transformation 555

transplantation 181

trastuzumab 788, 1504

treatment duration 966

trends 1269, 1296

TrkA 322

tumour antigens 1202

tumour cell 1497

tumour cell contamination 1326

tumour immunity 181

tumour markers 21, 425

tumour microenvironment 1131

tumour necrosis factor-related apoptosis-inducing ligand (TRAIL) 49

tumour regression 1020

tumour-associated macrophages 272

twins 520

type I insulin-like growth factor receptor 1220

typing 56

tyrosine kinase inhibitor 1483

UFT 159, 817

uranium miners 1280

uterine-sparing 1314

V600E *BRAF* 581

vaccination 896

validation 42

vascular endothelial growth factor (VEGF) 1013, 1611

vascular endothelial growth factor receptor-3 75

vascular endothelial growth factor-C 75

venous infiltration 1050

vessel 1

vimentin 1258

vinflunine 1161

vinorelbine 788

viral load 1384

virus-like particles 1459

vitamin D 1582

vitamin supplementation 677

vitronectin 1545

*von Hippel–Lindau* gene mutations 374

wartime 102

weekly administration 1005

weight loss 431

Wilms' tumour 541

ZD6126 722

zebularine 1701

